# Relationship between home care service use and changes in the care needs level of Japanese elderly

**DOI:** 10.1186/1471-2318-9-58

**Published:** 2009-12-21

**Authors:** Gohei Kato, Nanako Tamiya, Masayo Kashiwagi, Mikiya Sato, Hideto Takahashi

**Affiliations:** 1 Department of Health Services Research, Doctoral Program in Human Care Science, Graduate School of Comprehensive Human Sciences, University of Tsukuba, Ibaraki, Japan; 2 Department of Physical Therapy, Faculty of Health and Medical Care, Saitama Medical University, Saitama, Japan; 3 Tokyo Suginami Centre of Family Medicine, Kawakita General Hospital, Tokyo, Japan; 4 Epidemiology and Biostatistics, School of Medicine, University of Tsukuba, Tsukuba, Ibaraki, Japan

## Abstract

**Background:**

With the introduction of long-term care insurance (LTCI) in Japan, more home care services are available for the community-dwelling elderly. To deliver effective home care services, it is important to know the effects of service use. In this study, as the first step to determine this, we sought to describe different home service use in the sustained/improved group and deteriorated group in their care needs levels, and to report the relationship between the use of home care services and changes in care needs levels.

**Methods:**

The participants included 624 of a total of 1,474 users of LTCI services in one city in Japan. Home care service users were stratified into a 'lower care needs level subgroup' and a 'higher care needs level subgroup' based on the baseline care needs level. Simple statistical comparison and multiple logistic regression analyses in which the change in care needs level was set as a dependent variable were performed. Gender, age, and baseline care needs level were designated as control variables. Home based services were treated as independent variables. In this study, home care services consisted of home help, home bathing services, a visiting nurse, home rehabilitation, nursing home daycare, health daycare, loan of medical devices, respite stay in a nursing home, respite stay in a health care facility, respite stay in a sanatorium-type medical care facility, and medical management by a physician.

**Results:**

In the lower care needs level subgroup, age (OR = 1.04, CI, 1.01-1.08), use of respite stay in a nursing home (OR = 2.55; CI, 1.43-4.56), and the number of types of long-term care services (OR = 1.33; CI, 1.02-1.74) used during an 11 month period were significantly related to a deterioration of the user's care needs level. In the higher care needs level subgroup, use of medical management by a physician (OR = 6.99; CI, 1.42-41.25) was significantly related to a deterioration of the user's care needs level. There were no home based services significantly related to sustaining or improving the user's care needs level.

**Conclusion:**

There were different home service use in two groups (the sustained/improved group and the deteriorated group). Respite stay in a nursing home service use and more types of service use were related to experiencing a deterioration of care needs level in lower care needs level community-dwelling elderly persons in Japan. Further, medical management by a physician service was related to experiencing a deterioration of care needs level in higher care needs level community-dwelling elderly persons.

## Background

As a result of aging populations, the number of disabled elderly has become a major issue in most countries [1]. To address this issue, the Japanese government introduced a long-term care insurance (LTCI) system in 2000. The LTCI system seeks provide long-term care services including home based services to support the growing number of community-dwelling elderly persons and their families, as in other countries [1]. Researching the effectiveness of home care services on community-dwelling elderly persons has become important in preventing deterioration of the level of disability, not only for the day-to-day lives of the elderly, but also for the government in managing scarce health and social resources. One strategy for addressing the shortage of resources is increased standardization of care with performance indicators as measurements of quality [2,3]. Therefore, identifying the effect of home care services would contribute to standardization of and improvement in the quality of home care services.

Although the effectiveness of home care services has been revealed to some extent with the recent introduction of randomized control trial studies, it has been difficult to assure the quality of care research [4], and consequently there is inconsistent evidence in support of the effectiveness of home care services [5]. As a result, the effectiveness of home care services on recipients is still controversial. Some studies have reported positive effects of home care services including reductions in functional decline [6-10], mortality rates [11-15], institutionalization rates [8,10,12,16-18] and costs of care [19]. Other studies, however, have reported that home care services are ineffective in reducing functional decline [8], mortality rates [5,7] and institutionalization rates [5,9,20] in community-dwelling elderly persons.

Various effects have been produced by different types of home care service programs. The Japanese LTCI system is a multidimensional service program that provides social and medical services [21]. A report suggests that the utilization of home based services is more closely related to the needs of caregivers than the care needs level of the users [1]. Although multidimensional services and care needs levels are included in home based services studies, they have not been thoroughly studied [22,23]. Also, a small number of related studies in Japan [24,25] set care needs level as a main outcome, but those studies targeted only the mildly disabled elderly and not the severely disabled. There is still insufficient evidence regarding the effectiveness of components of home based services on care needs level. As a first step to determine the effect of service use on care needs level, an observational study to identify the relationship between home based service and changes in care needs level is necessary. We conducted a study to describe the differences in home service use between the sustained or improved group and the deteriorated group in their care needs level and to report the relationship between the use of home care services and changes in care needs levels. The user's care needs level was scaled according to the national standard on 'care needs level.' Multiple logistic regression analysis was applied to identify the relationship between service use and change in the care needs level, controlling for baseline confounding factors such as gender, age, and the baseline care needs level.

## Methods

The LTCI data were derived from a city 100 km west of Tokyo, Japan. The population on 1 October 2005 was 52,572 and the proportion of older people (aged 65 years or over) was 20.0%. This proportion is similar to the average in Japan (20.1%). The participants were all individuals who received long-term care services under the LTCI system in April 2005, and therefore healthy older people who did not receive long-term care services were not included in the study. The data was secondary data collected for the 11 months between April 2005 and February 2006. This data includes the LTCI service users' gender, age, monthly care needs level and monthly monetary amount of LTCI service utilization (details are reported elsewhere [26]).

The initial study population was 1,474 persons who were approved for LTCI in a period of 11 months. Since the purpose of this study was to describe the differences in home service use between the sustained or improved group and the deteriorated group in their care needs level, the LTCI service users (n = 624) who continued living in the community for 11 months were enrolled in this study as the main subjects of analysis. The LTCI service users who did not use home care service continuously 11 months (n = 229), of which 51 users were in the community at the baseline but received institutional care service in the following 10 months, were treated as discontinuous users. The remaining LTCI service users (n = 621) who did not register for the LTCI services at the baseline (n = 278) or were institutionalized at the baseline (n = 343) were excluded from this study.

The main outcome indicator was a 'change in the care needs level.' Care needs levels ranged from 0 to 5. Care needs levels were determined by the local government through a predetermined process. A trained local government official visits the home to evaluate nursing care needs using a questionnaire on current physical and mental status (73 items) and use of medical procedures (12 items) [27]. A government computer program classifies each applicant into one of six levels of dependency care after the evaluation. Finally, the Nursing Care Needs Certification Board, consisting of social and health services experts appointed by a mayor, determines whether initial care needs level are appropriate [27]. Care needs level 0 (assistance required) is intended for preventive services. The other five care needs levels (care required) range from lowest (care needs level 1) to the most severe (care needs level 5) needs.

The main subjects were divided into two groups: the group whose care needs level was sustained or improved (hereinafter, the SI group) and the group whose care needs level deteriorated (hereinafter, the D group). The change in the care needs level was calculated by subtracting the baseline care needs level from the level in the last month of care. If a user's change in care needs level was calculated to be 0 or <0, the change in the care needs level was defined as a sustained or improved care needs level (SI group). In the same manner, users whose change in care needs level was 1 or > 1 were defined as having a deterioration in care needs level (D group). To evaluate each component of home care service on the user's change in care needs level, gender, age, and home care service use were compared in the SI group and the D group. Home care services included home help, home bathing services, a visiting nurse, home rehabilitation, nursing home daycare, health daycare, loan of medical devices, respite stay in a nursing home, respite stay in a health care facility, respite stay in a sanatorium-type medical care facility, medical management by a physician and the number of types of services used during the 11 months. More detailed explanations are provided in the literature [21]. To define the service use variables, the monthly monetary amounts of home care service utilization were investigated for 11 months. If the monthly monetary amounts of the service utilization in a month were > 0 then the monthly service use data were scored one, and they were summed. Following this process, the service use variables were dichotomized (service use or not). They were recognized as 'service use' if the total monthly services use data were 1 or over.

Ethical considerations were examined in accordance with Japanese epidemiological guidelines for secondary data analysis. Our use of the data was approved by the city after we submitted a formal application for accessing data and explaining the purpose and data to be used. A pledge was also made to take maximum care in handling the data and to treat all data anonymously and in random order to prevent personal information from being revealed in the course of the study. Ethics approval was obtained from the University of Tsukuba Ethical Committee in Japan.

### Statistical analysis

The users were divided into two subgroups up to their baseline level of care to exclude the confounding effects of the physical and mental condition of the users. In Japan, the long-term care users who were higher than care needs level 3 were more likely to be institutionalized than those in care needs level 2 or lower [28]. The lower care needs level subgroup was set as users whose care needs levels were 0, 1, and 2, and the higher care needs level subgroup was set as users whose care needs levels were 3, 4, and 5.

The following statistical procedures were carried out to identify the relationship between the use of services and the change in the care needs level. First, home care service use and basic characteristics such as gender, age, and baseline care needs level were described for both the SI and the D groups. Second, simple statistical tests were carried out to compare basic characteristics and service use between the SI and the D groups. The metric and numeric variables were analyzed by an unpaired t-test and Wilcoxon rank-sum test, respectively. The categorical variables were analyzed using a χ^2^-test or Fisher's exact test. Third, multiple logistic regression analysis using stepwise variable selection method (inclusion and exclusion criteria = 20%) was adapted to build final models of the change in the care needs level. The change in care needs level was set as a dependent variable. Any services with p-value < 0.25 in simple statistical tests were imputed as independent variables based on the Hosmers and Lemeshow's screening criteria [29]. Age, gender, and the baseline care needs level were input as control variables in this analysis. The Hosmer-Lemeshow test and c-statistic were used to indicate the goodness-of-fit statistics of the models. The Kendall correlation coefficients among significant variables were analyzed to investigate the effect of collinearity.

To gain greater understanding of the results, three main sub-analyses were conducted. First, the duration of service use was compared between the SI and the D groups using the Wilcoxon rank-sum test. Service use durations were imputed as independent variables (0 to 11 months) into the final logistic model to estimate their effect instead of dichotomized variables (service use or not). The service use duration was calculated as the accumulated number of months in which a service was used during 11 months. Second, the effect of discontinuous users who stopped using home care service was investigated. The change in care needs level of discontinuous users (n = 229) was compared to that of the main subjects (n = 624) and stratified by baseline care needs level using the χ^2^-test. Service use by discontinuous users was compared between the SI and the D groups using the χ^2^-test or Fisher's exact test. Third, since this study was descriptive and used secondary data, it was difficult to set up the primary endpoint. Hence, post hoc statistical power analysis was conducted to evaluate the study sample size.

The statistical analysis was performed using PC-SAS. The significance level was set at < 0.05. The G*power version3.0.10 was applied for calculating statistical power (1-β error probability) [30]. The effect size was set at 0.30 for medium size [31].

## Results

Four hundred ninety-two of 624 users were in the lower care needs level subgroup and 132 were in the higher care needs level subgroup. Eighty-seven (18%) users in the lower care needs level and 16 (12%) users in the higher care needs level subgroup were recognized as D group, respectively. The baseline characteristics are shown in Table 1. The age variable in the lower care needs level subgroup had significantly different baseline characteristic between the SI and the D groups.

**Table 1:** Characteristics of service users by sustained or improved vs. deteriorated groups (n = 624)

Lower care needs level	Change in care needs level			
	Sustained or Improved (SI group)	Deteriorated (D group)		
*Variable*	n = 405	n = 87		
	Mean ± SD	Mean ± SD	t-value	p-value
Age^††^	80.3 ± 8.5	83.7 ± 7.7	3.50	< 0.001*
	n (%)	n (%)	χ^2^	p-value

Gender^†^				
Male	127 (31)	25 (29)		
Female	278 (69)	62 (71)	0.23	0.631
Baseline care needs level^†††^				
0	100 (25)	21 (24)		
1	226 (56)	44 (51)		
2	79 (20)	22 (25)		0.436

**Higher care needs level**	Change in care needs level			
	Sustained or Improved (SI group)	Deteriorated (D group)		
***Variables***	n = 116	n = 16		
	Mean ± SD	Mean ± SD	t-value	p-value

Age^††^	83.2 ± 10.6	83.5 ± 11.0	0.10	0.922
	n (%)	n (%)	χ^2^	p-value

Gender^†^				
Male	34 (29)	5 (31)		
Female	82 (71)	11 (69)	0.03	0.870
Baseline care needs level^†††^				
3	56 (48)	10 (63)		
4	35 (30)	6 (38)		
5	25 (22)	0 (0)		0.118

* p < 0.05

† χ^2^test

†† unpaired t-test

††† Wilcoxon rank-sum test

The results of simple statistical tests for lower care needs level subgroup and higher care needs level subgroup are shown in Table 2 and Table 3, respectively. In the lower care needs level subgroup, the D group used significantly more respite stay in a nursing home (48% vs. 20%, p < 0.001), nursing home daycare (79% vs. 67%, p = 0.029), and respite stay in sanatorium-type medical care facilities (2% vs. 0%, p = 0.031) and a greater number of types of care services during the 11 months (p < 0.001) than the SI group (Table 2). In the higher care needs level subgroup, the D group used significantly more medical management services by a physician (38% vs. 13%, p = 0.021) than the SI group (Table 3). There were no home based services that were used significantly more by the SI group than by the D group in both the lower and higher care needs level subgroups.

**Table 2:** Service use and change in care needs levels in the lower care needs level subgroup (n = 492)

	Change in care needs level			
	Sustained or Improved (SI group) n = 405	Deteriorated (D group) n = 87		
*Service use*	n (%)	n (%)	χ^2^	p-value
Home help^†^	128 (32)	30 (34)	0.27	0.602
Home bath service^††^	5 (1)	1 (1)		0.948
Visiting nurse^†^	27 (7)	9 (10)	1.43	0.232
Home rehabilitation^††^	5 (1)	1 (1)		0.948
Nursing home daycare^†^	273 (67)	69 (79)	4.79	0.029*
Health daycare^†^	72 (18)	11 (13)	1.35	0.246
Loan of devices^†^	129 (32)	30 (34)	0.23	0.634
Respite stay in a nursing home^†^	79 (20)	42 (48)	31.96	< 0.001*
Respite stay in health care facilities^††^	17 (4)	5 (6)		0.526
Respite stay in sanatorium-type medical care facilities^††^	0 (0)	2 (2)		0.031*
Medical management by a physician^††^	6 (1)	3 (3)		0.201
				
Number of kinds of care services used^†††^				
0	4 (1)	4 (5)		< 0.001*
1	170 (42)	19 (22)		
2	146 (36)	29 (33)		
3	68 (17)	18 (21)		
4	11 (3)	14 (16)		
5	5 (1)	2 (2)		
6	1 (0)	1 (1)		
Mean (SD)	1.83 (0.93)	2.33 (1.25)		

* p < 0.05

† χ^2^test

†† Fisher's exact test

††† Wilcoxon rank-sum test

**Table 3:** Service use and change in care needs levels in the higher care needs level subgroup (n = 132)

	Change in the care needs level			
	Sustained or Improved (SI group) n = 116	Deteriorated (D group) n = 16		
*Service use*	n (%)	n (%)	χ^2^	p-value
Home help^†^	47 (41)	8 (50)	0.52	0.473
Home bath service^††^	24 (21)	4 (25)		0.746
Visiting nurse^††^	29 (25)	7 (44)		0.137
Home rehabilitation^††^	4 (3)	1 (6)		0.482
Nursing home daycare^†^	73 (63)	9 (56)	0.27	0.606
Health daycare^††^	12 (10)	4 (25)		0.106
Loan of devices^††^	86 (74)	13 (81)		0.760
Respite stay in a nursing home^†^	52 (45)	7 (44)	0.01	0.935
Respite stay in health care facilities^††^	4 (3)	0 (0)		1.000
Respite stay in sanatorium-type medical care facilities^††^	2 (2)	1 (6)		0.324
Medical management by a physician^††^	15 (13)	6 (38)		0.022*
				
Number of kinds of care services used^†††^				
0	3 (3)	0 (0)		0.113
1	11 (9)	1 (6)		
2	29 (25)	3 (19)		
3	32 (28)	3 (19)		
4	26 (22)	5 (31)		
5	11 (9)	1 (6)		
6	4 (3)	2 (13)		
7	0 (0)	1 (6)		
Mean (SD)	3.00 (1.18)	3.75 (1.65)		

* p < 0.05

† χ^2 ^test

†† Fisher's exact test

††† Wilcoxon rank-sum test

Multivariate adjusted ORs and 95% CI for the covariates are shown in Table 4. The final model was built by stepwise multiple logistic regression analysis. Gender, age, and the baseline care needs level were included as control variables in both models. In the lower care needs level subgroup, visiting nurse, nursing home daycare, health daycare, respite stay in a nursing home, respite stay in sanatorium-type medical care facilities, medical management by a physician and number of kinds of care services used were imputed as independent variables. In higher care needs level subgroup, visiting nurse, health daycare, medical management by a physician and number of kinds of services used were imputed as independent variables. As a result, in the lower care needs level subgroup, higher age (OR = 1.04; CI, 1.01-1.08), more use of respite stay in a nursing home (OR = 2.55; CI, 1.39-4.56), and a greater number of types of care services during the 11 months (OR = 1.33; CI, 1.02-1.74) were included in the final model and significantly related to a deteriorating care needs level. The results of the Hosmer-Lemeshow test was p = 0.23, and the c-statistic was 0.69. In the higher care needs level subgroup, medical management by a physician (OR = 6.99; CI, 1.42-41.25) and a lower care needs level in the first month (OR = 0.16; CI, 0.03-0.53) were included in the final model. The use of a visiting nurse (OR = 4.65; CI, 0.99-19.98) and health day care service (OR = 3.01; CI, 0.63-13.53) were not significantly related but remain in the final model. The result of the Hosmer-Lemeshow test was p = 0.99 and the c-statistic was 0.83. The Kendall correlation coefficients among the independent variables imputed in the final models were less than 0.50.

**Table 4:** Multivariate adjusted ORs and 95% CI for care needs levels deteriorating

*Lower care needs level subgroup*		
Variable	Adjusted OR	95% CI
Gender		
Female	1.09	0.63-1.91
Age	1.04*	1.01-1.08
Baseline care needs level	0.89	0.60-1.31
Respite stay in a nursing home	2.55*	1.43-4.56
Number of kinds of care services used	1.33*	1.02-1.74

Goodness-of-fit statistics: χ^2 ^= 11.15; p = 0.23^†^, c-statistic = 0.69		

***Higher care needs level subgroup***		
Variable	Adjusted OR	95%CI

Gender		
Female	1.76	0.48-7.47
Age	0.99	0.94-1.06
Baseline care needs level	0.16*	0.03-0.53
Visiting nurse	4.65	0.99-19.98
Health daycare	3.01	0.63-13.53
Medical management by a physician	6.99*	1.42-41.25

Goodness-of-fit statistics: χ^2 ^= 1.43; p = 0.99^†^, c-statistic = 0.83		

* p < 0.05

† Hosmer-Lemeshow test

As the results of sub-analysis, we determined first that the D group in the lower care needs subgroup had significantly longer durations of respite stay in nursing homes (mean +/- SD 2.3 +/- 3.4 vs. 1.0 +/- 2.6, p < 0.01) and in sanatorium-type medical care facilities (mean +/- SD 0.950 +/- 2.6 vs. 0.00 +/- 0.0, p < 0.01) than the SI group. The D group in higher care needs subgroup had significantly longer durations of medical management by a physician (mean +/- SD 3.40 +/- 4.9 vs. 1.1 +/- 3.0, p = 0.01) than the SI group. The adjusted OR of the duration of the respite stay in a nursing home service use was 1.10 (CI, 1.01-1.18) in the lower care needs level subgroup. Gender, age baseline care needs level were used for the adjustment. The duration of respite stay in a nursing home and number of kinds of care services used were forced into the model. The adjusted OR of the duration of the medical management by a physician service use was 1.21 (CI, 1.03-1.44) in the higher care needs level subgroup. Gender, age baseline care needs level were used for the adjustment, the duration of visiting nurse, health daycare and medical management by a physician were forced into the model.

Second, there was no significant difference in the change in care needs level between main subjects and discontinuous users in both care needs level subgroups. In lower care needs subgroup, the proportions of the D group in main subjects and discontinuous users were 18% (n = 87) and 22% (n = 31), respectively. In the same manner, in the higher care needs subgroup, they were 12% (n = 16) and 16% (n = 14), respectively. With regard to service use by discontinuous users, no significant differences were found between the SI and the D groups in both care needs level subgroups.

Third, the post hoc statistical powers for the lower care needs level subgroup and higher care needs level subgroup were 0.99 and 0.93, respectively.

## Discussion

The comparison of the two groups defined by outcome stratified by baseline care needs levels revealed the difference in home service use between the two groups (SI and D groups).

The use of respite stay in a nursing home, increased types of service use, and higher age were significantly related to a worsening care needs level (D group) in the lower care needs level subgroup. Further, use of medical management by a physician and a lower baseline care needs level were significantly related to a worsening care needs level in the higher care needs level subgroup.

Our study is descriptive, and therefore we cannot be certain of causal relationships. We can conjecture, however, that there are two possibilities to explain why some service use showed a significant relationship with a deteriorating care needs level: (1) user's potential to deteriorate, not effect of service use; and (2) the effect of service use.

As to the potential of respite service users to deteriorate, first, we need to consider the possibility that respite service users had more risk factors of declining care needs level. Poorer health, poorer physical and cognitive functions are identified as risk factors for mortality in community dwelling frail older people [15]. Secondary, as respite service is reported to be used by recipients with stressed caregivers [32,33], and that relates to poorer quality of care and causes adverse effects on recipients [34], respite service users may have had such the characteristics before service use.

Though our study cannot indicate the effect, the reported effect of respite service use in previous studies itself is also still controversial. The adverse effect of respite service users' Activity of Daily Living (ADL) functional performance and sleep rhythms were observed in past studies [35-37]. However, some literature reported that there were no adverse effect from respite stay service on older users and users with dementia in the standard program unit [38,39] or a special unit for people with dementia [40].

It may be necessary to focus more on the content of respite service to investigate the effect of respite service on changes in the care needs level. We need to consider the lack of the perspective of the respite care recipients like rehabilitation because respite stay service has been developed with the aim of easing the burdens of caregivers. This is supported by the fact that numerous publications evaluate the effects of respite care focusing on outcomes for caregivers not care recipients [41].

In sum, we did not control detailed characteristic of users, such as comorbid conditions etc, care givers' variables and the content of services. It is difficult to explain the causal effects which might underlie the relationship between service use and decline of care needs levels. Further research investigating the detailed characteristics of the users and the content of respite service is necessary.

A greater number of types of care services used during the 11 months was related to a deterioration of the user's care needs level in the lower care needs level subgroup. Utilization of greater number of services might reflect complicated care needs as a potential to deteriorate. The types of services used increased when the user's care needs level deteriorated because the users were allowed to use more services offered by the LTCI system. In contrast to our study result, a Japanese study reported use of only one service or more than two types of services was not statistically related to a deterioration of care needs level [24]. The difference with our study might be caused by stratification of the numbers of types of services used. Kikuzawa et al. [24] categorized users into two groups, one using one type of care service and another using two or more types of care services. However, we treated the number of types of care services as ordinal variables. Hence, this difference might be caused by a difference in statistical sensitivity. More detailed research is required to elucidate this difference.

Medical management by a physician service use were related to a deterioration in care needs level by users in the higher care needs level subgroup. One of the possible causes of deterioration might be the users' severe mental or physical disabilities. According to the literature, disease burden is one of the main factors for deteriorating of function among the elderly [42]. Moreover, it was reported that elderly persons in poor health gain no benefit from home visiting programs [5]. In contrast, home geriatric assessment in well-functioning community-dwelling elderly showed a benefit from preventive in-home geriatric assessment strategies [8,42]. Since we did not control for physical or mental impairments or comorbid condition which would affect the care needs level change and particularly for medical management by a physician, more investigation into the detailed characteristics of the service users might contribute to uncovering this mechanism.

Concerning the baseline confounding factors for deteriorating care needs level, higher age is an understandable factor for deterioration of physical or mental functioning of users in the lower care needs level subgroup. It has been suggested that the young-old population gain greater benefit from multidimensional geriatric assessment and multiple follow-up home visit services than the old-old population [8]. In the higher care needs level subgroup, the reason that a higher baseline care needs level contributed to a deterioration of the user's care needs level might be that the users whose care needs levels were five had no possibility to experience a deterioration of care needs level. Therefore, a seemingly baseline care needs level was related to a deteriorating of the user's care needs level in the higher care needs level subgroup.

There is also a possibility of selection bias. Our main result excludes discontinuous users. Therefore, these findings may be limited to LTCI users who have lived in the community continuously for 11 months. However, the exclusion of the discontinuous users may not significantly change our findings. This is because in the discontinuous group, the proportions of the SI and D groups were not significantly different and also, no services were significantly related to the change in care needs level.

The length of our study may also limit our findings. Eleven months may be a relatively short period to observe the relationship between home services use and changes in the care needs level of community-dwelling elderly persons. However, our findings might have some degree of consistency because our study used the change in care needs level as an outcome indicator, not an indicator such as mortality rate, which fundamentally requires a longer follow-up for evaluation. Further, the sample size of users who deteriorated was tolerated to conduct the statistical analysis for detecting medium effect size.

## Conclusion

There are differences in service use between the sustained or improved care needs level group and the deteriorated care needs level group. We concluded that respite stay in nursing homes and more types of services used were related to a deterioration of the users with lower care needs levels. Medical management by a physician service was related to deterioration in care needs level in higher care needs level subgroup who might be severely disabled.

## Competing interests

The authors declare that they have no competing interests.

## Authors' contributions

GK carried out structuring the study design, statistical analysis, interpreting the data, and drafting the manuscript. NT supervised all the process as the corresponding author: participated in the design of the study,statistical analysis, interpretation  of  the  data, and  helped  to finalize the  manuscript.  MK participated in designing this study, acquiring the data, and structuring the data set. MS carried out special advice to structure the data set. HT helped to create the SAS program to perform the statistical analysis. All authors read and approved the final manuscript.

## Pre-publication history

The pre-publication history for this paper can be accessed here:

http://www.biomedcentral.com/1471-2318/9/58/prepub
